# Cancer Risk in Children of Mothers With Epilepsy and High-Dose Folic Acid Use During Pregnancy

**DOI:** 10.1001/jamaneurol.2022.2977

**Published:** 2022-09-26

**Authors:** Håkon Magne Vegrim, Julie Werenberg Dreier, Silje Alvestad, Nils Erik Gilhus, Mika Gissler, Jannicke Igland, Maarit K. Leinonen, Torbjörn Tomson, Yuelian Sun, Helga Zoega, Jakob Christensen, Marte-Helene Bjørk

**Affiliations:** 1 Department of Clinical Medicine, University of Bergen, Bergen, Norway; 2 National Centre for Register-Based Research, School of Business and Social Sciences, Aarhus University, Aarhus, Denmark; 3 National Centre for Epilepsy, Oslo University Hospital, Oslo, Norway; 4 Department of Neurology, Haukeland University Hospital, Bergen, Norway; 5 Department of Knowledge Brokers, Finnish Institute for Health and Welfare, Helsinki, Finland; 6 Institute of Molecular Medicine and Surgery, Karolinska Institute, Stockholm, Sweden; 7 Department of Clinical Neuroscience, Karolinska Institute, Stockholm, Sweden; 8 Department of Neurology, Aarhus University Hospital, Aarhus, Denmark; 9 Department of Clinical Medicine, Aarhus University, Aarhus, Denmark; 10 Department of Clinical Epidemiology, Aarhus University Hospital, Aarhus, Denmark; 11 School of Population Health, Faculty of Medicine and Health, University of New South Wales, Sydney, Australia; 12 Centre of Public Health Sciences, Faculty of Medicine, University of Iceland, Reykjavik, Iceland; 13 Department of Clinical Neurophysiology, Haukeland University Hospital, Bergen, Norway

## Abstract

**Importance:**

Women with epilepsy are recommended high doses of folic acid before and during pregnancy owing to risk of congenital anomalies associated with antiseizure medications. Whether prenatal exposure to high-dose folic acid is associated with increases in the risk of childhood cancer is unknown.

**Objective:**

To assess whether high-dose folic acid supplementation in mothers with epilepsy is associated with childhood cancer.

**Design, Setting, and Participants:**

Observational cohort study conducted with nationwide registers in Denmark, Norway, and Sweden from 1997 to 2017. Analyses were performed during January 10, 2022, to January 31, 2022. Mother-child pairs were identified in medical birth registers and linked with information from patient, prescription, and cancer registers, as well as with sociodemographic information from statistical agencies, and were categorized by maternal diagnosis of epilepsy. The study population consisted of 3 379 171 children after exclusion of 126 711 children because of stillbirth or missing or erroneous values on important covariates.

**Exposures:**

Maternal prescription fills for high-dose folic acid tablets (≥1 mg daily) between 90 days before pregnancy start and birth.

**Main Outcomes and Measures:**

First onset of childhood cancer at younger than 20 years. Cox proportional hazards models were used to calculate adjusted hazard ratios with corresponding 95% CIs, adjusted for potential confounders. Cumulative incidence at aged 20 years was used as a measure of absolute risk.

**Results:**

The median age at the end of follow-up in the study population of 3 379 171 children was 7.3 years (IQR, 3.5-10.9 years). Among the 27 784 children (51.4% male) born to mothers with epilepsy, 5934 (21.4%) were exposed to high-dose folic acid (mean dose, 4.3 mg), with 18 exposed cancer cases compared with 29 unexposed, producing an adjusted hazard ratio of 2.7 (95% CI, 1.2-6.3), absolute risk if exposed of 1.4% (95% CI, 0.5%-3.6%), and absolute risk if unexposed of 0.6% (95% CI, 0.3%-1.1%). In children of mothers without epilepsy, 46 646 (1.4%) were exposed to high-dose folic acid (mean dose, 2.9 mg), with 69 exposed and 4927 unexposed cancer cases and an adjusted hazard ratio of 1.1 (95% CI, 0.9-1.4; absolute risk, 0.4% [95% CI, 0.3%-0.5%]). There was no association between children born to mothers with epilepsy who were prenatally exposed to antiseizure medications, but not high-dose folic acid, and an increased risk of cancer (absolute risk, 0.6%; 95% CI, 0.2%-1.3%).

**Conclusions and Relevance:**

Prenatal exposure to high-dose folic acid was associated with increased risk of cancer in children of mothers with epilepsy.

## Key Points

**Question:**

Is prenatal exposure to high-dose folic acid (≥1 mg daily) associated with risk of cancer in children born to mothers with epilepsy?

**Findings:**

In this cohort study of 3 379 171 children, compared with no high-dose folic acid use in pregnancy for mothers with epilepsy, use among those with epilepsy was associated with an increased risk of cancer in their children.

**Meaning:**

Findings suggest that cancer risk in children should be considered in the risk-benefit analysis of folic acid supplementation for pregnant women with epilepsy.

## Teaser

This cohort study uses nationwide register data from 3 Nordic countries to examine the association between risk of childhood cancer and high-dose folic acid supplementation in mothers with epilepsy.

## Introduction

Women with epilepsy are often recommended high doses of folic acid (up to 5 mg daily) before and during pregnancy to reduce the risk of congenital anomalies associated with prenatal exposure to antiseizure medication (ASM).^1^ Folate is an important vitamin for synthesis and repair of nucleic acids, and supplementation during pregnancy to women in general (0.4-0.8 mg of folic acid daily) has been shown to reduce the risk of neural tube defects in the child.^2^ However, concern has been raised about a possible cancer risk with folic acid supplementation, possibly through altered DNA methylation.^3,4^ High levels of folate may promote progression of neoplastic lesions and induce oxidative stress.^5,6^ Folate deficiency could impair synthesis and repair of DNA and hence increase the risk of cancer.^7^

Cancer is the second most frequent cause of death in children in high-income countries, and the incidence is increasing.^8^ There are few known risk factors apart from ionizing radiation, chemotherapy, and high maternal age.^9,10,11,12,13,14,15^ Data on the association between prenatal exposure to folic acid and childhood cancer derive from studies of folic acid dose levels commonly used by mothers in the general population, but little is known regarding prenatal exposure to higher doses.^16,17,18,19,20,21^ Because ASMs use can be an indication for high-dose folic acid supplementation in pregnancy, it is essential to consider their role in the potential association between childhood cancer and high-dose folic acid. In this study, we used nationwide register data from 3 Nordic countries to examine maternal prescription fill for high-dose folic acid (≥1 mg daily) during pregnancy and risk of cancer in the child, considering the potential role of maternal ASM use.

## Methods

### Study Population

For this cohort study, we identified 3 505 882 singletons born in Denmark (1997-2017), Norway (2005-2017), and Sweden (2006-2017) from the national medical birth registers. These countries have personal identification numbers enabling individual linkage between national registers providing detailed health and socioeconomic information on all inhabitants (eAppendix in the Supplement).^22^ Data were collected in the Nordic Register-Based Study of Antiepileptic Drugs in Pregnancy (SCAN-AED) collaboration project.^23^

**Figure 1.**
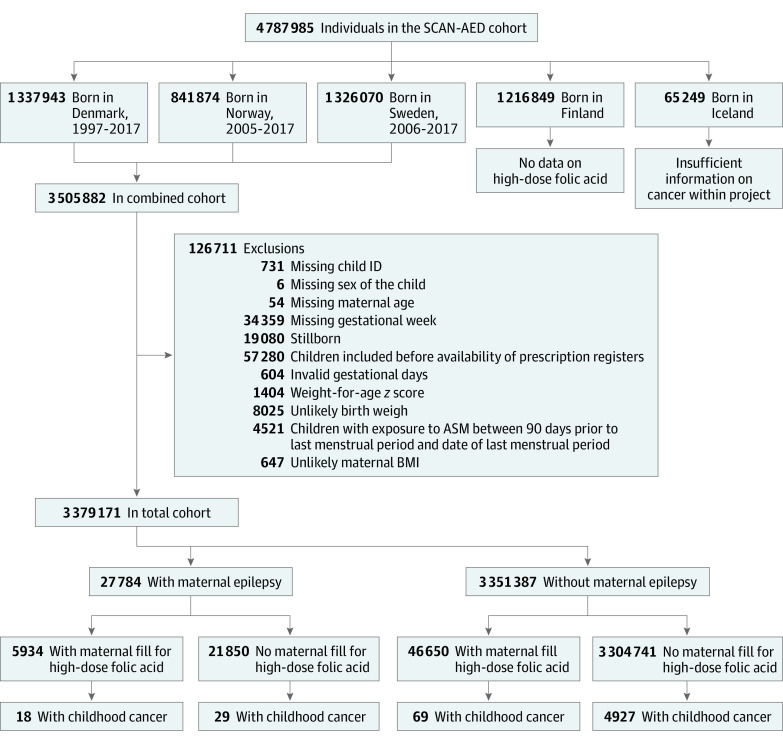
Flowchart of the Study Population Selection of study and control population between mothers with and without epilepsy who did and did not fill a prescription of high-dose folic acid. ASM indicates antiseizure medication; BMI, body mass index (calculated as weight in kilograms divided by height in meters squared); and SCAN-AED, Nordic Register-Based Study of Antiepileptic Drugs in Pregnancy.

The final study population consisted of 3 379 171 mother-child pairs, including 27 784 children of mothers with epilepsy. An overview of excluded pairs (n = 126 711 [3.7%]) is shown in Figure 1.

This study was approved by all relevant authorities in each country. Informed consent was not obtained (eTable 1 in the Supplement) because this was a purely register-based study with anonymized data from mandatory registers and no informed consent procedures. We used the Strengthening the Reporting of Observational Studies in Epidemiology (STROBE) reporting guideline. A complete and detailed overview for all the included covariates is available in eTable 12 in the Supplement.

### High-Dose Folic Acid

We defined prenatal exposure to high-dose folic acid as at least 1 filled prescription of either 1 mg or 5 mg folic acid supplement (Anatomical Therapeutic Chemical code B03BB01)^24^ between 90 days before the first day of the last menstrual period (hereinafter “pregnancy start”) and birth.^1,25^ The mean folic acid dose (in milligrams) was calculated according to the number of pills dispensed per day after pregnancy start and until birth, requiring at least 1 prescription.

### Childhood Cancer Diagnoses

We used the national cancer registers to identify cases of childhood cancer. These registers are considered close to complete in all 3 countries.^26^ The *International Classification of Diseases for Oncology, Third Edition* contains information on cancer topography and morphology and was used to define cancers as malignant according to behavioral code value greater than or equal to 3.^27^ Categorization of childhood cancers was according to the *International Classification of Childhood Cancer, Third Edition*.^28^

### Maternal Epilepsy and ASM Exposure

Maternal epilepsy was defined as a recorded diagnosis of epilepsy any time before date of delivery, using *International Statistical Classification of Diseases and Related Health Problems, Tenth Edition (ICD-10*) codes G40 to G41.^29^ We defined prenatal exposure to ASMs as at least 1 prescription fill from pregnancy start until birth, using Anatomical Therapeutic Chemical code N03, N05BA09, or S01EC01. To reduce misclassification of ASM exposure, we excluded mother-child pairs in which the mothers had an ASM prescription fill only during the 90 days before the date of the last menstrual period and the date of last menstrual period, but none during later the stages of pregnancy. We identified the most frequently used monotherapy ASMs during the pregnancies: carbamazepine, lamotrigine, levetiracetam, and valproate. Separate variables for these ASMs in combination with any other ASM were made. We classified the less frequently prescribed ASM monotherapies of topiramate, oxcarbazepine, clonazepam, and phenobarbital as “other monotherapy.” ASM polytherapy exposure was defined as prescription fills of more than 1 type of ASM.

### Covariates

We applied covariates according to known risk factors for childhood cancer that could also be associated with high-dose folic acid supplementation.^10^ Maternal covariates included age, educational level, smoking status, and body mass index (calculated as weight in kilograms divided by height in meters squared) recorded at the first prenatal appointment and previous birth of a child with a major congenital anomaly. Number of hospital admissions during the year before pregnancy start and until birth was used as a proxy for maternal comorbidity. We also included maternal diagnoses of diabetes (gestational diabetes or type 1 or 2 diabetes), tuberous sclerosis, and cancer before pregnancy.

Child covariates included any major congenital anomaly (*ICD-10* codes Q00-Q89) or chromosomal abnormality (*ICD-10* codes Q90-Q99), both recorded until 1 year after birth. Congenital anomalies are associated with cancer in childhood and adulthood.^30^

### Statistical Analysis

#### Primary Analyses

We applied a Cox proportional hazards regression model to report hazard ratios (HRs) and corresponding 95% CIs with child age as the time scale. Statistical significance was determined via 95% CI. Children were followed up from birth until date of first cancer diagnosis or censored at the date of death, emigration, 20th birthday, or the end of follow-up (December 31, 2017), whichever came first. Birth year, source country, and sex of the child were applied as strata within each model. Assumption of proportional hazards was evaluated for all variables and models.

In the primary analysis, we examined prenatal exposure to high-dose folic acid and childhood cancer in children of mothers with epilepsy, with corresponding incidence rates per 100 000 person-years including a cumulative incidence at age 20 years as an absolute measure of risk. We adjusted for maternal age and educational level, ASM prescription fill, number of hospitalizations, smoking, body mass index, and previous birth of a child with a major congenital anomaly. We used multiple imputation by chained equations to handle missingness on maternal educational level (122 510 of 3 379 171; 3.6%), smoking (280 934; 8.3%), and body mass index (978 863; 29.0%), with 20 imputations.^31^ Analyses were performed with Stata version 16 (StataCorp) from January 10, 2022, to January 31, 2022.

#### Secondary Analyses

We reran the primary analysis stratified by maternal ASM prescription fill during pregnancy to explore ASM as a variable and calculated the interaction effect between ASM and high-dose folic acid.

By constructing cumulative incidence curves separately for exposed and unexposed children of mothers with or without epilepsy, we made a graph comparing the cumulative incidence with corresponding 95% CIs of cancer in children of mothers with or without epilepsy who were exposed or unexposed to high-dose folic acid. Death was treated as a competing event and age was used as the time scale. The graph line and corresponding CIs were smoothed owing to data protection legislation.

The primary analysis was repeated with end of follow-up at 10 years of age to investigate whether the risk was higher for the youngest children. We compared the frequency of the 3 most common cancer types according to prenatal exposure to high-dose folic acid and maternal epilepsy status and calculated the HRs. The increasing mean daily dose of filled prescriptions of folic acid after pregnancy start (categorized into >0 mg to <4 mg and >4 mg, and >0 mg to <3 mg and >3 mg, respectively) and cancer in the child were investigated compared with unexposed children.

#### Sensitivity Analyses

We investigated childhood cancer and prenatal exposure to maternal prescription fill for any ASM and for different ASM types and compared them with those of children of mothers not filling a prescription for any ASM. We calculated the adjusted HRs (aHRs) for childhood cancer by using a maternal diagnosis of epilepsy as the exposure and compared them with those of children of mothers without epilepsy. This calculation was performed regardless of folic acid prescription to have a sufficient number of exposed cancer cases.

Factors that could modify the risk of childhood cancer were separately excluded from the primary analysis in post hoc analyses: maternal cancer, tuberous sclerosis,^32^ diabetes,^33,34^ and major congenital anomalies or chromosomal abnormalities in children.^30^ We also removed any maternal prescription fills for carbamazepine or valproate after pregnancy start and until birth because prenatal use of these ASMs leads to a higher risk of congenital anomalies.^35^

## Results

Among the 3 379 171 children in the study, we identified 27 784 born to mothers with epilepsy (0.8%), with median age 7.3 years (IQR, 3.5-10.9 years) at the end of follow-up, 13 501 (48.6%) female, and 14 283 (51.4%) male. A total of 5934 children (21.4%) were exposed to high-dose folic acid. Among 3 351 387 children of mothers without epilepsy (99.2%), 46 646 (1.4%) were exposed to high-dose folic acid (Table 1). The median follow-up time in the entire cohort was 7.3 years (IQR, 3.5-10.9 years). The estimated mean daily folic acid dose for mothers with epilepsy was higher than that for mothers without epilepsy (4.3 mg [SD = 4.1] vs 2.9 mg [SD = 2.9]) (Table 1 and eTable 2 in the Supplement). Most mothers with epilepsy with prescription fills for folic acid also filled ASM prescriptions (5531 of 5934 [93.2%]). Among mothers with epilepsy who used ASM, 48.4% (5195 of 10 726) did not fill high-dose folic acid prescriptions (Table 1). The mean doses of lamotrigine, levetiracetam, carbamazepine, and valproate were highest among mothers with epilepsy prescription filling for high-dose folic acid (Table 1).

**Table 1.** Population Characteristics of 3 379 171 Children Born in Denmark, Norway, and Sweden Stratified on Maternal Epilepsy and Prenatal Exposure to High-Dose Folic Acid

Characteristic	No. (%)			
	No maternal epilepsy (3 351 387 [99.2%])		Maternal epilepsy (27 784 [0.8%])	
	No high-dose folic acid	High-dose folic acid	No high-dose folic acid	High-dose folic acid
Total^a^	3 304 741 (98.6)	46 646 (1.4)	21 850 (78.6)	5934 (21.4)
Countries^a^				
Denmark	1 272 265 (98.4)	8457 (0.7)	10 657 (0.8)	1654 (0.1)
Norway	759 367 (98.7)	4105 (0.5)	4940 (0.6)	1045 (0.1)
Sweden	1 273 109 (96.7)	34 084 (2.6)	6253 (0.5)	3235 (0.2)
Birth year^a^				
1997-2001	313 290 (98.1)	4278 (1.3)	1340 (0.4)	377 (0.1)
2002-2006	526 355 (98.3)	4977 (0.9)	3378 (0.6)	809 (0.2)
2007-2011	1 125 287 (97.8)	16 263 (1.4)	7510 (0.7)	2119 (0.2)
2012-2016	1 118 014 (97.6)	16 745 (1.5)	8013 (0.7)	2221 (0.2)
2017	221 795 (97.2)	4383 (1.9)	1609 (0.7)	408 (0.2)
Characteristics of the mother				
Maternal age, mean (SD), y	30.3 (5.1)	31.4 (5.5)	29.6 (5.3)	30.6 (5.0)
Maternal educational level^b^				
Compulsory	490 881 (14.9)	7931 (17.0)	5551 (25.4)	1105 (18.6)
Pre-university	1 452 762 (44.0)	22 429 (48.1)	9422 (43.1)	2838 (47.9)
College/university	792 322 (24.0)	8912 (19.1)	4314 (19.7)	1367 (23.1)
Postgraduate	448 353 (13.6)	5947 (12.7)	1996 (9.1)	531 (8.9)
Missing	120 423 (3.6)	1427 (3.1)	567 (2.6)	93 (1.6)
Smoking at the beginning of pregnancy^b^				
No	2 711 554 (82.1)	38 394 (82.3)	16 829 (77.0)	4890 (82.5)
Yes	318 094 (9.6)	4248 (9.1)	3612 (16.5)	616 (10.4)
Missing	275 093 (8.3)	4004 (8.6)	1409 (6.5)	428 (7.2)
BMI^b^				
Mean (SD)	24.6 (5.1)	25.5 (5.3)	25.0 (5.4)	25.2 (5.0)
<30	2 050 366 (62.0)	30 258 (64.9)	13 932 (63.8)	3877 (65.4)
≥30	292 140 (8.8)	6444 (13.8)	2559 (11.7)	732 (12.3)
Missing	962 235 (29.1)	9944 (21.3)	5359 (24.5)	1325 (22.3)
Maternal diabetes^b^				
Type 1 or 2 diabetes	30 144 (0.9)	1196 (2.6)	582 (2.7)	111 (1.9)
Gestational diabetes	77 705 (2.4)	1493 (3.2)	655 (3.0)	141 (2.4)
Maternal hospital admissions^b^				
0	3 020 239 (91.4)	38 035 (81.5)	19 154 (87.7)	5201 (87.6)
1	266 604 (8.1)	7473 (16.0)	2204 (10.1)	655 (11.0)
≥2	17 876 (0.5)	1138 (2.4)	492 (2.2)	78 (1.3)
Maternal medication				
Folic acid dose, mean (SD), mg	0	2.9 (2.9)	0	4.3 (4.1)
Any ASM^b^	6045 (0.2)	819 (1.8)	5195 (23.8)	5531 (93.2)
ASM polytherapy (yes/no)^c^	20 (0.3)	6 (0.7)	137 (2.6)	223 (4.0)
ASM monotherapy^c^				
Valproate	196 (3.2)	39 (4.8)	337 (6.5)	572 (10.3)
Lamotrigine	2655 (43.9)	451 (55.1)	2176 (41.9)	1931 (34.9)
Levetiracetam	16 (0.3)	6 (0.7)	490 (9.4)	348 (6.3)
Carbamazepine	201 (3.3)	47 (5.7)	529 (10.2)	1025 (18.5)
Topiramate	193 (3.2)	13 (1.6)	80 (1.5)	93 (1.7)
Oxcarbazepine	33 (0.5)	11 (1.3)	289 (5.6)	194 (3.5)
Phenobarbital	102 (1.7)	7 (0.8)	26 (0.5)	17 (0.3)
Clonazepam	374 (6.2)	19 (2.3)	166 (3.2)	49 (0.9)
Dose of different ASM before and during pregnancy, mean (SD), mg				
Valproate	79.6 (73.4)	119.2 (123.2)	151.5 (162.1)	223.0 (177.5)
Lamotrigine	164.4 (164.3)	246.0 (206.6)	301.3 (248.7)	450.9 (363.9)
Carbamazepine	85.3 (99.3)	143.8 (130.0)	225.5 (180.3)	284.5 (205.3)
Levetiracetam	52.8 (53.0)	173.1 (162.3)	312.2 (248.4)	433.8 (347.1)
Child characteristics^b^				
Male sex	1 697 735 (51.4)	23 620 (50.6)	11 261 (51.5)	3022 (50.9)
Female sex	1 607 006 (48.6)	23 026 (49.4)	10 589 (48.5)	2912 (49.1)
Gestational age, mean (SD), wk	39.3 (2.0)	38.9 (2.3)	39.0 (2.2)	39.0 (2.3)
Infant birth weight, mean (SD), g	3497.4 (590.8)	3397.1 (656.9)	3432.8 (621.6)	3428.8 (643.3)
Major congenital anomaly	165 024 (5.0)	2457 (5.3)	1339 (6.1)	422 (7.1)

Abbreviations: ASM, antiseizure medication; BMI, body mass index (calculated as weight in kilograms divided by height in meters squared).

^a^ Expressed as number (rowwise percentage) of women with or without high-dose folic acid exposure.

^b^ Expressed as number (columnwise percentage) of women with or without high-dose folic acid exposure.

^c^ Columnwise percentages among the total number of any ASM usage in each subgroup.

The incidence rate of cancer in children of mothers with epilepsy who filled prescriptions for high-dose folic acid was 42.5 (95% CI, 26.8-67.5) per 100 000 person-years (n = 18) compared with 18.4 (95% CI, 12.8-26.5) per 100 000 person-years (n = 29) in children of mothers with epilepsy who did not fill folic acid prescriptions. In children of mothers without epilepsy, the aHR of cancer in 69 children exposed to high-dose folic acid was 1.1 (95% CI, 0.9-1.4; absolute risk, 0.4% [95% CI, 0.3%-0.5%]) compared with 4927 children unexposed to high-dose folic acid (absolute risk, 0.4; 95% CI, 0.4-0.4) (Table 2). The absolute risk of cancer was 1.5% (95% CI, 0.5%-3.5%) (Table 2) in exposed children of mothers with epilepsy, with an aHR for cancer of 2.7 (95% CI, 1.2-6.3), compared with children of mothers with epilepsy who were unexposed to high-dose folic acid (absolute risk, 0.6%; 95% CI, 0.3%-1.1%).

**Table 2.** Association Between Maternal Epilepsy, Filled Prescription of High-Dose Folic Acid, and Risk of Childhood Cancer in the Offspring^a^

Maternal epilepsy	High-dose folic acid	Live births	Childhood cancer cases	Incidence rate per 100 000 person-years (95% CI)	Cumulative incidence at 20 y (95% CI)^b^	Crude HR (95% CI)	aHR 1 (95% CI)^c^	aHR 2 (95% CI)^d^	aHR 3 (95% CI)^e^
Yes	Yes	5934	18	42.5 (26.8-67.5)	1.5 (0.5-3.6)	2.4 (1.3-4.5)	2.4 (1.3-4.4)	2.5 (1.1-5.8)	2.7 (1.2-6.3)
	No	21 850	29	18.4 (12.8-26.5)	0.6 (0.3-1.1)	1 [Reference]	1 [Reference]	1 [Reference]	1 [Reference]
No	Yes	46 646	69	20.0 (15.8-25.4)	0.4 (0.3-0.5)	1.1 (0.9-1.4)	1.1 (0.9-1.4)	1.1 (0.9-1.4)	1.1 (0.9-1.4)
	No	3 304 741	4927	18.9 (18.4-19.5)	0.4 (0.4-0.4)	1 [Reference]	1 [Reference]	1 [Reference]	1 [Reference]

Abbreviations: aHR, adjusted hazard ratio; aHR 1, first model with basic adjustment; aHR 2, second model including adjustment for antiseizure medication prescription fill; aHR 3, fully adjusted model.

^a^ Birth year, sex of the child and source country were applied as stratum for all models.

^b^ The cumulative incidence at aged 10 years was 0.4 (95% CI, 0.2-0.6) for maternal epilepsy yes and high-dose folic acid yes; 0.2 (95% CI, 0.1-0.3) for maternal epilepsy yes and high-dose folic acid no; 0.2 (95% CI, 0.1-0.3) for maternal epilepsy no and high-dose folic acid yes; and 0.2 (95% CI, 0.2-0.2) for maternal epilepsy no and high dose folic acid no.

^c^ Adjustment for maternal age and educational level.

^d^ Including adjustment for antiseizure medication exposure.

^e^ Including adjustment for maternal body mass index, prior births with major congenital anomalies, smoking during pregnancy, and number of hospitalizations.

### Secondary Analyses

In children of mothers with epilepsy who filled prescriptions for ASM, a 3.0-fold (95% CI, 1.1-fold to 7.9-fold) increased risk of childhood cancer associated with exposure to high-dose folic acid was observed. Women with epilepsy who filled prescriptions for ASM but not for high-dose folic acid did not have an increased risk of cancer in their offspring (absolute risk, 0.6%; 95% CI, 0.2%-1.3%). There were insufficient data to estimate the cancer risk associated with high-dose folic acid use in children of mothers with epilepsy who had no filled prescriptions for ASM (Table 3). There was no statistically significant interaction between ASM and high-dose folic acid among children born to mothers with or without epilepsy (eTable 3 in the Supplement).

**Table 3.** Association Between Maternal Epilepsy, Maternal Prescription Fill for Antiseizure Medication (ASM), High-Dose Folic Acid, and Risk of Childhood Cancer in the Offspring^a^

Maternal epilepsy	Maternal ASM	High-dose folic acid	Live births	Incidence rate per 100 000 person-years (95% CI)	Cumulative incidence at 20 y (95% CI)^b^	Crude HR (95% CI)	aHR 1 (95% CI)^c^	aHR 2 (95% CI)^d^
Yes	Yes	Yes	5531	43.0 (26.8-69.2)	1.4 (0.5-3.1)	2.9 (1.1-7.5)	2.9 (1.1-7.7)	3.0 (1.1-7.9)
		No	5195	19.1 (9.5-38.1)	0.6 (0.2-0.13)	1 [Reference]	1 [Reference]	1 [Reference]
	No	Yes	403	NA	NA	NA	NA	NA
		No	16 655	18.2 (11.8-27.9)	0.6 (0.3-1.1)	1 [Reference]	1 [Reference]	1 [Reference]
No	Yes	Yes	819	NA	NA	NA	NA	NA
		No	6045	23.0 (11.5-46.0)	0.4 (0.1-1.0)	1 [Reference]	1 [Reference]	1 [Reference]
	No	Yes	45 827	19.7 (15.4-25.1)	0.4 (0.3-0.5)	1.1 (0.8-1.4)	1.1 (0.8-1.4)	1.1 (0.8-1.4)
		No	3 298 696	18.9 (18.4-19.5)	0.4 (0.4-0.4)	1 [Reference]	1 [Reference]	1 [Reference]

Abbreviations: aHR, adjusted hazard ratio; aHR 1, first model with basic adjustment; aHR 2, second model including adjustment for antiseizure medication prescription fill; NA, not available.

^a^ Birth year, sex of the child and source country were applied as stratum for all models.

^b^ The cumulative incidence at age 10 years was 0.4 (95% CI, 0.2-0.6) for maternal epilepsy yes, maternal ASM yes, and high-dose folic acid yes; 0.1 (95% CI, 0.1-0.3) for maternal epilepsy yes, maternal ASM yes, and high-dose folic acid no; 0.2 (95% CI, 0.1-0.3) for maternal epilepsy yes, maternal ASM no, and maternal high-dose folic acid no; 0.2 (95% CI, 0.1-0.6) for maternal epilepsy no, maternal ASM yes, and maternal high-dose folic acid no; 0.2 (95% CI, 0.1-0.3) for maternal epilepsy no, maternal ASM no, and maternal high-dose folic acid yes; and 0.2 (95% CI, 0.2-0.2) for maternal epilepsy no, maternal ASM no, and maternal high-dose folic acid no.

^c^ Adjustment for maternal age and educational level.

^d^ Including adjustment for maternal body mass index, prior births with major congenital anomalies, smoking during pregnancy, and number of hospitalizations.

**Figure 2.**
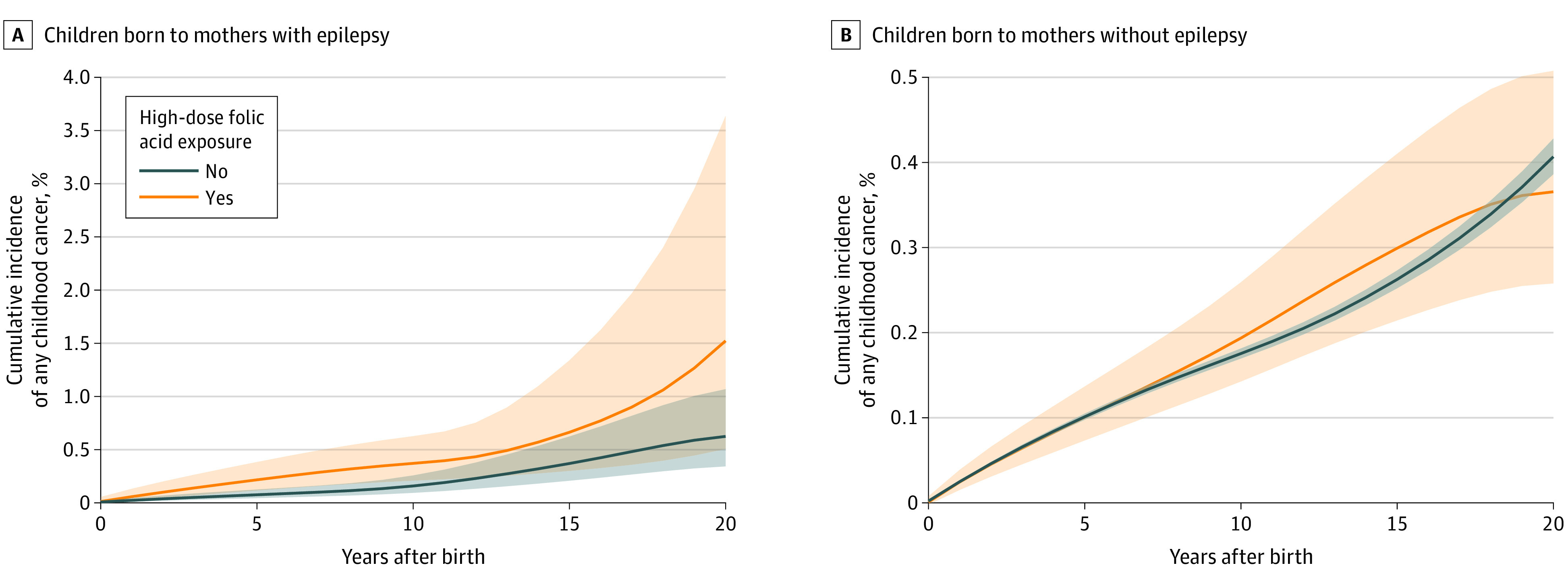
Cumulative Incidence of Childhood Cancer Cumulative incidence of first onset of childhood cancer recorded from birth until 20 years of age with or without maternal prescription fill for high-dose folic acid for mothers with or without a diagnosis of epilepsy. The graph lines were smoothed owing to the Danish Data Protection Act to prevent identification of individuals.

For children younger than 10 years born to mothers with epilepsy, the cumulative cancer incidence was higher in exposed children than unexposed ones (Figure 2), with an aHR of 3.2 (95% CI, 1.2-8.7) (eTable 4 in the Supplement). In all exposure groups, leukemia was the most common type of cancer, followed by lymphoma and central nervous system tumors (eTable 5 in the Supplement). The aHR for leukemia among children born to mothers with epilepsy who filled a prescription for high-dose folic acid was 7.3 (95% CI, 1.5-35.2) (eTable 6 in the Supplement). We did not have sufficient exposed cancer cases to report HRs for other cancer types.

Children of mothers with epilepsy with prenatal exposure to average prescribed daily dose greater than 4 mg folic acid had an aHR for cancer of 3.4 (95% CI, 1.1-10.7) compared with unexposed children of mothers with epilepsy, whereas the aHR was 2.9 (95% CI, 1.2-7.2) in children exposed to doses below 4 mg. There was no increased risk of cancer in children born to mothers without epilepsy within the same groups of estimated daily dose of folic acid (eTable 7 in the Supplement). When the same calculations based on average prescribed daily dose greater than 0 to less than 3 mg and at least 3 mg were performed, the results remained unchanged compared with the estimates with a 4-mg cutoff (eTable 8 in the Supplement).

### Sensitivity Analyses

The aHR of cancer in children exposed to ASM was 1.5 (95% CI, 1.1-2.1) compared with that of children unexposed to any ASM regardless of maternal epilepsy and prescription fill for high-dose folic acid. These estimates were similar for children of mothers with or without epilepsy (aHR, 1.6 [95% CI, 0.9-2.8] vs 1.4 [95% CI, 0.8-2.6]). There were no risk differences between specified ASM monotherapies or polytherapy because all point estimates overlapped the 95% CI of the overall association between ASM and cancer in children of mothers with or without epilepsy (eTable 9 in the Supplement).

Maternal epilepsy was not associated with childhood cancer (aHR, 1.0; 95% CI, 0.7-1.4) (eTable 10 in the Supplement). In the primary analysis, removal of mothers with cancer before pregnancy (n = 454), tuberous sclerosis (n = 23), or diabetes (n = 1292, including gestational diabetes) did not change the findings between high-dose folic acid and cancer in the child. Excluding mothers with epilepsy with any prescription for carbamazepine or valproate (n = 3407) only slightly attenuated the aHR (aHR, 2.4; 95% CI, 0.9-6.5). Excluding children with major congenital anomalies (n = 1761) or chromosomal abnormalities (n = 68) who were born to mothers with epilepsy did not change the risk estimates (eTable 11 in the Supplement).

## Discussion

In this multinational population-based study of more than 3 million pregnancies, we identified a 2.7-fold increased risk of cancer in children of mothers with epilepsy who immediately before or during pregnancy filled a prescription for high-dose folic acid (≥1 mg daily) compared with children of mothers with epilepsy who did not fill such a prescription. The estimates were consistent after adjustment for potential confounders, were unchanged after removal of children with other possible risk factors for childhood cancer, and could not readily be explained by maternal use of ASM. However, because ASM was used by almost the entire cohort of women with epilepsy filling prescriptions for high-dose folic acid (5531 of 5934 [93.2%]) (Table 1), it was not possible to completely rule out that maternal use of ASM in pregnancy or other characteristics inherent in mothers with epilepsy who used high-dose folic acid could partly or fully explain the finding. Pathologies of inherent diseases other than those we were able to control for might still be associated with the cancer risk.

The increased risk associated with high-dose folic acid was also found when stratified for ASM use. However, we found no association between maternal prescription fills for any specific ASM and childhood cancer. Removing mothers with any prescription fills for carbamazepine and valproate was not associated with the point estimate. Hence, these 2 ASMs were not important effect modifiers for the cancer association. Maternal epilepsy was not associated with an increased risk of cancer among the study children. The most frequent childhood cancer types in children among mothers with epilepsy who filled prescriptions for high-dose folic acid did not differ from the distribution in the general population.^8^

We did not find any increased risk of cancer in exposed children of mothers without epilepsy. A previous population-based study from the Norwegian Birth Register did not find an increased risk of cancer in children in the general population after maternal use of folic acid in doses up to 0.6 mg daily during pregnancy.^36^ Ecologic studies in the US and Canada described a reduction of childhood cancer incidence after implementation of folic acid fortification in foods, but with estimated folic acid doses below 0.4 mg daily.^20,21,37^ Other studies have shown a reduced risk of childhood central nervous system tumors^17^ and acute lymphoblastic leukemia,^16,18,19^ based on self-reported folic acid usage after doses of approximately 0.4 mg daily, and no certain supplementation greater than 0.8 mg.

In mothers who filled prescriptions for high-dose folic acid, the mean calculated daily dose after pregnancy start and until birth was higher among those with epilepsy (4.3 mg) than without it (2.9 mg). Although an elevated risk of cancer was observed in children born to mothers with epilepsy who were exposed to increasing mean doses of folic acid, the differences between those with more than 4 mg daily compared with less than 4 mg daily were not significant. The mean calculated ASM doses were higher among mothers with epilepsy with a filled prescription for high-dose folic acid compared with those with epilepsy with no prescription, which may imply that an increasing dose of ASM plays a role in carcinogenesis among children born to mothers with epilepsy, possibly through an interaction between ASM and folic acid in high doses. However, we did not identify a statistically significant interaction between ASM and high-dose folic acid, but the statistical power of this analysis was limited.

### Strengths and Limitations

A major strength of this study is the use of population-based information from mandatory and nationwide health and administrative registers, providing a sufficient sample size to examine associations between rare combinations of exposures and outcomes. The exposure information was based on filled prescriptions, eliminating recall bias. The likelihood of misclassification of cancers is negligible owing to the high validity of the data from the national cancer registers.^26^

There are some limitations to this study. A filled prescription of high-dose folic acid does not guarantee that mothers took the medication every day.^38^ This affects the calculation for the mean daily folic acid dose. An increasing mean daily dose can also depict the length of supplementation rather than the daily dose taken. We did not have information on serum levels of folic acid to assess maternal intake, any over-the-counter supplements, and dietary intake. Despite the large study sample, we did not have enough exposed childhood cancers to report estimates for children of mothers with epilepsy who filled prescriptions for high-dose folic acid but not for ASM or risk estimates for cancer subtypes except for leukemia.

## Conclusions

In this cohort study, an association was found between increased risk of cancer among children of mothers with epilepsy and maternal use of high-dose folic acid. In contrast, prenatal exposure to high-dose folic acid was not associated with an increased risk of childhood cancer in children born to mothers without epilepsy.

Results of this study should be considered when the risks and benefits of folic acid supplements for women with epilepsy are discussed and before decisions about optimal dose recommendations are made. Because of the combined use of ASM and folic acid in high doses in mothers with epilepsy, future studies should investigate possible etiologic mechanisms between folic acid and ASM exposure in pregnancy and the risk of cancer.

## Notes

Supplement. **eAppendix.** Description of Data Sources **eTable 1.** Ethical Approvals **eTable 2.** Mean Dose Delivered of High-Dose Folic Acid Among Different Maternal Comorbidities **eTable 3.** Interaction Between Antiseizure Medication and High-Dose Folic Acid in Mothers With and Without Epilepsy **eTable 4.** Risk of Childhood Cancer During the First 10 Years of Life in Children Born to Mothers With and Without Epilepsy Filling Prescriptions for High-Dose Folic Acid **eTable 5.** High-Dose Folic Acid and Type of Childhood Cancer **eTable 6.** Risk of Leukemia in Children Born to Mothers With and Without Epilepsy Filling Prescriptions for High-Dose Folic Acid **eTable 7.** Association Between Cancer in the Child and Average Delivered Dose of Folic Acid in Mothers With and Without Epilepsy **eTable 8.** Association Between Cancer in the Child and Average Delivered Dose of Folic Acid in Mothers With and Without Epilepsy, Based on Estimated Mean Dose of Folic Acid **eTable 9.** Prescription Fill for Antiseizure Medication and Risk of Childhood Cancer **eTable 10.** Effect of Maternal Epilepsy on Risk of Childhood Cancer **eTable 11.** Restrictions **eTable 12.** Definition of Included Covariates
eReferences
