# Supplementary material for: Cancer Risk in Children of Mothers With Epilepsy and High-Dose Folic Acid Use During Pregnancy
Source: JAMA Neurol. 2022 Sep 26;79(11):1130–8. doi: 10.1001/jamaneurol.2022.2977 (PMC9513705; doi:10.1001/jamaneurol.2022.2977)
Supplement: Supplement. — eAppendix. Description of Data Sources eTable 1. Ethical Approvals eTable 2. Mean Dose Delivered of High-Dose Folic Acid Among Different Maternal Comorbidities eTable 3. Interaction Between Antiseizure Medication and High-Dose Folic Acid in Mothers With and Without Epilepsy eTable 4. Risk of Childhood Cancer During the First 10 Years of Life in Children Born to Mothers With and Without Epilepsy Filling Prescriptions for High-Dose Folic Acid eTable 5. High-Dose Folic Acid and Type of Childhood Cancer eTable 6. Risk of Leukemia in Children Born to Mothers With and Without Epilepsy Filling Prescriptions for High-Dose Folic Acid eTable 7. Association Between Cancer in the Child and Average Delivered Dose of Folic Acid in Mothers With and Without Epilepsy eTable 8. Association Between Cancer in the Child and Average Delivered Dose of Folic Acid in Mothers With and Without Epilepsy, Based on Estimated Mean Dose of Folic Acid eTable 9. Prescription Fill for Antiseizure Medication and Risk of Childhood Cancer eTable 10. Effect of Maternal Epilepsy on Risk of Childhood Cancer eTable 11. Restrictions eTable 12. Definition of Included Covariates eReferences [file jamaneurol-e222977-s001.pdf]

Supplementary Online Content

Vegrim HM, Dreier JW, Alvestad S, et al. Cancer risk in children of mothers with epilepsy and high-dose folic acid use during pregnancy. *JAMA Neurol*. Published online September 26, 2022. doi:10.1001/jamaneurol.2022.2977

eAppendix. Description of Data Sources

eTable 1. Ethical Approvals

eTable 2. Mean Dose Delivered of High-Dose Folic Acid Among Different Maternal Comorbidities

eTable 3. Interaction Between Antiseizure Medication and High-Dose Folic Acid in Mothers With and Without Epilepsy

eTable 4. Risk of Childhood Cancer During the First 10 Years of Life in Children Born to Mothers With and Without Epilepsy Filling Prescriptions for High-Dose Folic Acid

eTable 5. High-Dose Folic Acid and Type of Childhood Cancer

eTable 6. Risk of Leukemia in Children Born to Mothers With and Without Epilepsy Filling Prescriptions for High-Dose Folic Acid

eTable 7. Association Between Cancer in the Child and Average Delivered Dose of Folic Acid in Mothers With and Without Epilepsy

eTable 8. Association Between Cancer in the Child and Average Delivered Dose of Folic Acid in Mothers With and Without Epilepsy, Based on Estimated Mean Dose of Folic Acid

eTable 9. Prescription Fill for Antiseizure Medication and Risk of Childhood Cancer

eTable 10. Effect of Maternal Epilepsy on Risk of Childhood Cancer

eTable 11. Restrictions

eTable 12. Definition of Included Covariates

eReferences.

This supplementary material has been provided by the authors to give readers additional information about their work.

© 2022 Vegrim HM et al. *JAMA Neurology*.

eAppendix. Description of Data Sources

Data from several nationwide, mandatory health registers were collected to perform this study. Below is a simple flowchart showing which and how the different registers were used.

Medical birth registers (MBR)

MBR contains information on all births in mothers including national identification number, age, smoking, maternal parity, pregnancy and delivery diagnoses and interventions, offspring anomalies, birth outcome, gestational age, weight at birth, Apgar score. It is mandatory for health care providers to report to this register, and information is collected from medical records from the prenatal, delivery and neonatal care. The Medical Birth Register of Norway was founded in 1967, and the Danish and Swedish ones in 1973.

In Norway, the use of folic acid supplement (any dose) pre-conception and/or during pregnancy is recorded in the birth register.

Prescription registers (PDR)

PDR contains data on dispensed prescriptions from pharmacies. This includes ATC-codes, drug strength, total defined daily dose dispensed, package size, and dispensing date. FDR does not contain information about over-the-counter medicines and medicines used during in-patient hospital stays. The Danish Registry of Medicinal Product Statistics contains information from 1995 and onwards, the Norwegian Prescription Database from 2004 and the Swedish Prescribed Drug Register from July 2005. The Norwegian and Danish Prescription Databases also contain information about the indication for reimbursement.

National patient registers (NPR)

NPR contains information about diagnosis according to ICD-10 codes. The Norwegian Patient Registry contains information on all persons treated at hospitals as well in private clinics if they are reimbursed by the public health system. This information has been linked to the personal, national identification numbers since 2008. The Danish National Hospital Register contains information from hospital stays from 1977. ICD codes for psychiatric treatment are available from 1969 from the Psychiatric Central Research Register. From 1995, also data from somatic and psychiatric outpatient contacts are available. The Swedish National patient register includes all in-patient care in Sweden from 1987. The outpatient visits including psychiatric care from both private and public caregivers were included from 2001.

National statistical agencies (NSA)

Each of the Nordic countries have national statistical agencies containing information on socioeconomic and demographic data on all inhabitants. These data contain yearly information on household income, education, occupation and demographical data on emigration and death. Most of the variables are digitized and follow international standards (ISCO-88 for occupation, ISCED2011 for education). Hence harmonization between the registers was feasible.

Cancer registers (CR)

The Danish CR was established in 1941, in Norway in 1951 and in Sweden in 1958. It is mandatory for healthcare-providers to report information on diagnosis, treatment, and cause of death to the national cancer-registers.

All the CRs contain information about age, sex, time of diagnosis, site and type of tumor, stage, or extent of the cancer, in addition to several other variables, this for the entire study-period. CR uses an international standard (ICD-O-3) which contains precise information on morphology and topography.

© 2022 Vegrim HM et al. *JAMA Neurology*.

eTable 1. Ethical Approvals

Ethical approvals were necessary to retrieve and manage data from the national social and health registers. This included approvals from the Data Protection Agency in Denmark; the Norwegian Inspectorate and the Regional Ethics Committee for Medical Research in Western Norway; and the Regional Ethical Board at Karolinska Institutet in Sweden. Below is an overview of approvals.

Ethical approvals from included countries

Country	Ethical approval	Data protection approvals	Data transfer agreement	Data protection agreement
Denmark	Not required for register-based research	Data protection agency	N/A	✓
Norway	✓	Data protection impact assessment	✓	✓
Sweden	✓	Local Data protection Officer	✓	✓

Abbreviations: N/A: Not applicable.
All register holders must approve of the project. Necessary approvals can change over time.
All data was exported to Denmark; thus, a data transfer agreement was not necessary.

eTable 2. Mean Dose Delivered of High-Dose Folic Acid Among Different Maternal Comorbidities

Maternal characteristics or comorbidities	Mean dose in mg (SD)
General population	3.1 (3.1)
General population without maternal epilepsy	2.9 (2.9)
Maternal epilepsy	4.3 (4.1)
Maternal use of ASM	4.3 (4.1)
Maternal use of ASM without epilepsy	3.1 (3.2)
Maternal use of VPA in combination with any ASM	4.4 (4.5)
Maternal use of LTG in combination with any ASM	4.1 (3.8)
Maternal diabetes mellitus	2.9 (3.1)
Maternal smoking during the start of pregnancy	2.6 (2.8)
Maternal BMI $\geq$30 kg/m^2	3.4 (3.5)

Abbreviations: ASM: Antiseizure medication; BMI: Body Mass Index; LTG: Lamotrigine; SD: Standard deviation; VPA: Valproic acid
The mean dose delivered of high dose folic acid in mg was calculated along with corresponding standard deviation for different maternal characteristics, comorbidities and comedications.

eTable 3. Interaction Between Antiseizure Medication and High-Dose Folic Acid in Mothers With and Without Epilepsy

Test for interaction between antiseizure medication and high dose folic acid on the risk of cancer in the offspring was performed in children to mothers with and without epilepsy. Level of significance was set to $p<0.05$. Test for interaction was based on the fully adjusted model as presented in Table 2 by adding an interaction term to the analysis.

Test for interaction between antiseizure medication (ASM) and high dose folic acid on cancer risk in children to mothers with and without epilepsy

Test for interaction	p-value
Children to mothers with epilepsy	0.76
Children to mothers without epilepsy	0.48

Abbreviations: ASM: Antiseizure medication.

© 2022 Vegrim HM et al. *JAMA Neurology*.

eTable 4. Risk of Childhood Cancer During the First 10 Years of Life in Children Born to Mothers With and Without Epilepsy Filling Prescriptions for High-Dose Folic Acid

The risk of childhood cancer during the first ten first years of follow-up after birth was investigated for children born to mothers with and without diagnosis of epilepsy, filling prescription for high dose folic acid before and during pregnancy. The risk of cancer was 3.2 [95% CI 1.2-8.7] in children during the first ten years of follow-up if they were born to mothers with epilepsy filling prescription of high dose folic acid, compared to mothers with epilepsy that did not have such a prescription.

Association between maternal epilepsy, filled prescription of high dose folic acid, and risk of childhood cancer in the offspring up to age 10 years

Maternal epilepsy	High dose folic acid	Live births	Incidence rate per 100 000 person-years, 95% CI	Crude HR, 95% CI	aHR1[a], 95% CI	aHR2[b], 95% CI	aHR3[c], 95% CI
Yes	Yes	4462	40.5 (24.4-67.2)	2.9 (1.4-6.0)	2.8 (1.3-5.7)	3.1 (1.1-8.4)	3.2 (1.2-8.7)
	No	15 750	14.6 (9.4-22.6)	1 [Reference]	1 [Reference]	1 [Reference]	1 [Reference]
No	Yes	34 264	20.0 (15.4-25.9)	1.1 (0.9-1.5)	1.1 (0.9-1.5)	1.1 (0.9-1.4)	1.1 (0.9-1.4)
	No	2 270 632	18.2 (17.7-18.8)	1 [Reference]	1 [Reference]	1 [Reference]	1 [Reference]

a) aHR1: Adjustment for maternal age and education
b) aHR2: Including adjustment for antiseizure medication exposure.
c) aHR3: Including adjustment for maternal BMI, prior births with congenital anomalies, smoking during pregnancy, number of hospitalizations.
Birth year, sex of the child and source country were applied as stratum for all models.

eTable 5. High-Dose Folic Acid and Type of Childhood Cancer

Occurrence (number and percentage) of the three most ICCC3 subgroups of cancer among children born to mothers with and without epilepsy filling for high dose folic acid. The three most common types of childhood cancers were also the most common types of cancer in children of mothers with epilepsy filling for high dose folic acid.

Frequency of cancer cases in different childhood cancer subgroups and fill for high dose folic acid

ICCC3 subgroups	No epilepsy		Epilepsy	
	No high dose folic acid	High dose folic acid	No high dose folic acid	High dose folic acid
Group I: Leukemia	1620 (30%)	28 (40%)	8 (30%)	8 (40%)

© 2022 Vegrim HM et al. *JAMA Neurology*.

Group II & III: Lymphoma and CNS tumors	1007 (20%)	14 (20%)	7 (30%)	4 (<25%)
Other childhood cancer subtypes	2300	27	15	6
Abbreviations: ICCC3: International Classification of Childhood Cancer 3rd edition.				

eTable 6. Risk of Leukemia in Children Born to Mothers With and Without Epilepsy Filling Prescriptions for High-Dose Folic Acid

We calculated the HR for the most common childhood cancer type, leukemia, in children to mothers with or without epilepsy filling for high dose folic acid (1mg or 5mg folic acid). There was not sufficient no. of exposed cancer cases to report HRs for other cancer subtypes.

Association between fill for high dose folic acid by mothers with and without epilepsy and risk of leukemia in the offspring

Maternal epilepsy	High dose folic acid	Live births	Cancer cases	Incidence rate per 100 000 person-years, 95% CI	Crude HR, 95% CI	aHR1[a], 95% CI	aHR2[b], 95% CI	aHR3[c], 95% CI
Yes	Yes	4462	8	18.9 (9.5-37.8)	4.2 (1.5-12.3)	4.2 (1.4-12.2)	6.3 (1.3-30.6)	7.3 (1.5-35.2)
	No	15 750	8	5.1 (2.5-10.2)	1 [Reference]	1 [Reference]	1 [Reference]	1 [Reference]
No	Yes	34 264	28	8.1 (5.5-11.8)	1.3 (0.9-1.9)	1.3 (0.9-1.9)	1.3 (0.9-1.9)	1.3 (0.9-1.9)
	No	2 270 632	1620	6.3 (6.0-6.6)	1 [Reference]	1 [Reference]	1 [Reference]	1 [Reference]

a) aHR1: Adjustment for maternal age and education
b) aHR2: Including adjustment for antiseizure medication exposure.
c) aHR3: Including adjustment for maternal BMI, prior births with congenital anomalies, smoking during pregnancy, number of hospitalizations.
Birth year, sex of the child and source country were applied as stratum for all models.

eTable 7. Association Between Cancer in the Child and Average Delivered Dose of Folic Acid in Mothers With and Without Epilepsy

The average delivered dose of folic acid was calculated based on prescription fills for 1 mg and 5 mg of folic acid, filled between 90 days prior to the date of last menstrual period and until birth. This was categorized into >0 mg to <4 mg daily and ≥4 mg daily. These categories were examined for mothers with and without epilepsy, comparing both groups with mothers that had no fill for high dose folic acid. We were unable to make more categories due to few cancer cases among the exposed children.

© 2022 Vegrim HM et al. *JAMA Neurology*.

**Association between average daily dose of folic acid and risk of cancer in the offspring to mothers with and without epilepsy**								
**Maternal epilepsy**	**Average daily dose of folic acid**	**Live births**	**Cancer cases**	**Incidence rate per 100 000 person-years, 95% CI**	**Crude HR, 95% CI**	**aHR1, 95% CI[a]**	**aHR2, 95% CI[b]**	**aHR3, 95% CI[c]**
Yes	>0 mg to <4 mg	3389	11	44.0 (24.3-79.4)	2.5 (1.2-5.1)	2.4 (1.2-4.9)	2.8 (1.1-6.8)	2.9 (1.2-7.2)
	No folic acid	21 850	29	18.4 (12.8-26.5)	1 [Reference]	1 [Reference]	1 [Reference]	1 [Reference]
	≥4 mg	2545	7	40.5 (19.3-85.0)	2.9 (1.1-7.4)	2.8 (1.1-7.1)	3.1 (1.0-9.4)	3.4 (1.1-10.7)
	No folic acid	21 850	29	18.4 (12.8-26.5)	1 [Reference]	1 [Reference]	1 [Reference]	1 [Reference]
No	>0 mg to <4 mg	35 307	54	20.7 (15.8-27.1)	1.1 (0.9-1.5)	1.1 (0.9-1.5)	1.1 (0.9-1.5)	1.1 (0.9-1.5)
	No folic acid	3 304 741	4874	19.0 (18.5-19.5)	1 [Reference]	1 [Reference]	1 [Reference]	1 [Reference]
	≥4 mg	11 339	14	17.8 (10.5-30.1)	1.0 (0.6-1.7)	1.0 (0.6-1.6)	1.0 (0.6-1.6)	1.0 (0.6-1.6)
	No folic acid	3 304 741	4874	19.0 (18.5-19.5)	1 [Reference]	1 [Reference]	1 [Reference]	1 [Reference]

a) aHR1: Adjustment for maternal age and education
b) aHR2: Including adjustment for ASM exposure.
c) aHR3: Including adjustment for maternal BMI, prior births with congenital anomalies, smoking during pregnancy, number of hospitalizations.
Birth year, sex of the child and source country were applied as stratum for all models.

eTable 8. Association Between Cancer in the Child and Average Delivered Dose of Folic Acid in Mothers With and Without Epilepsy, Based on Estimated Mean Dose of Folic Acid

The estimated mean dose in mothers with and without epilepsy were 4.3mg and 2.9mg, respectively. Therefore, we reran the analysis as presented in eTable 7, but here comparing use of mean daily prescribed dose of folic acid >0 to <3mg and ≥3mg. The numbers have for this table been rounded to closest ten due to close similarity with eTable 7 with the possibility to calculate difference with less than 5 exposed cancer cases.

**Association between average daily dose of folic acid and risk of cancer in the offspring to mothers with and without epilepsy, based on estimated mean dose folic acid.**								
**Maternal epilepsy**	**Average daily dose of folic acid**	**Live births**	**Cancer cases**	**Incidence rate per 100 000 person-years, 95% CI**	**Crude HR, 95% CI**	**aHR1, 95% CI[a]**	**aHR2, 95% CI[b]**	**aHR3, 95% CI[c]**

© 2022 Vegrim HM et al. *JAMA Neurology*.

Yes	>0 mg to <3 mg	3070	10	39.7 (20.7-76.3)	2.2 (1.0-4.7)	2.1 (1.0-4.6)	2.5 (1.0-6.6)	2.6 (1.0-6.9)
	No folic acid	21 850	30	18.4 (12.8-26.5)	1 [Reference]	1 [Reference]	1 [Reference]	1 [Reference]
	≥3 mg	2860	10	45.8 (23.8-88.0)	2.9 (1.3-6.7)	2.8 (1.2-6.4)	3.0 (1.1-8.3)	3.3 (1.2-9.4)
	No folic acid	21 850	30	18.4 (12.8-26.5)	1 [Reference]	1 [Reference]	1 [Reference]	1 [Reference]
No	>0 mg to <3 mg	33 660	50	20.8 (15.8-27.4)	1.1 (0.9-1.5)	1.1 (0.9-1.5)	1.1 (0.9-1.5)	1.1 (0.9-1.5)
	No folic acid	3 304 740	4870	18.9 (18.4-19.5)	1 [Reference]	1 [Reference]	1 [Reference]	1 [Reference]
	≥3 mg	12 990	20	17.9 (10.9-29.2)	1.0 (0.6-1.6)	1.0 (0.6-1.6)	1.0 (0.6-1.6)	1.0 (0.6-1.6)
	N/A	3 304 740	4870	18.9 (18.4-19.5)	1 [Reference]	1 [Reference]	1 [Reference]	1 [Reference]

a) aHR1: Adjustment for maternal age and education
b) aHR2: Including adjustment for ASM exposure.
c) aHR3: Including adjustment for maternal BMI, prior births with congenital anomalies, smoking during pregnancy, number of hospitalizations.
Birth year, sex of the child and source country were applied as stratum for all models.
Counts have been rounded to closest ten due to close similarity to counts presented in eTable 7.

eTable 9. Prescription Fill for Antiseizure Medication and Risk of Childhood Cancer

The risk of childhood cancers with maternal fills for ASM was examined. This was performed regardless of high dose folic acid use or not to be able to obtain a sufficient number of exposed cancer cases to report risk estimates.

Hazard rates (HR) of cancer in offspring of women with prescription fills for antiseizure-medication (ASM) regardless of high dose folic acid prescriptions, stratified for maternal epilepsy and types and combinations of antiseizure medications.

Type of ASM	Filled prescription of ASM	Incidence rates per 100 000 person years	Crude HR, 95% CI	aHR1*, 95% CI	aHR2**, 95% CI
Any ASM	Yes	29.00 (20.82-30.49)	1.47 (1.04-2.06)	1.46 (1.04-2.06)	1.49 (1.07-2.09)
	No	18.93 (18.41-19.47)	1 [Reference]	1 [Reference]	1 [Reference]
	Yes	30.69 (20.73-45.41)	1.54 (0.84-2.78)	1.48 (0.82-2.69)	1.56 (0.86-2.83)

© 2022 Vegrim HM et al. *JAMA Neurology*.

Hazard rates (HR) of cancer in offspring of women with prescription fills for antiseizure-medication (ASM) regardless of high dose folic acid prescriptions, stratified for maternal epilepsy and types and combinations of antiseizure medications.					
**Type of ASM**	**Filled prescription of ASM**	**Incidence rates per 100 000 person years**	**Crude HR, 95% CI**	**aHR1*, 95% CI**	**aHR2**, 95% CI**
Any ASM within mothers with epilepsy	No	18.58 (12.24-28.22)	1 [Reference]	1 [Reference]	1 [Reference]
Any ASM within mothers without epilepsy	Yes	25.49 (13.71-47.37)	1.36 (0.73-2.52)	1.34 (0.72-2.50)	1.42 (0.76-2.64)
	No	18.93 (18.41-19.47)	1 [Reference]	1 [Reference]	1 [Reference]
≥ 2 prescriptions of any ASM	Yes	28.11 (19.28-40.99)	1.39 (0.94-2.05)	1.39 (0.94-2.05)	1.40 (0.95-2.07)
	No	18.95 (18.42-19.48)	1 [Reference]	1 [Reference]	1 [Reference]
Any polytherapy	Yes	N/A[a]	N/A[a]	N/A[a]	N/A[a]
	No	18.49 (17.16-19.93)	1 [Reference]	1 [Reference]	1 [Reference]
Any monotherapy	Yes	26.89 (18.02-40.12)	1.36 (0.99-2.06)	1.35 (0.89-2.05)	1.36 (0.90-2.07)
	No	18.95 (18.43-19.49)	1 [Reference]	1 [Reference]	1 [Reference]
Valproate in combination with any ASM	Yes	35.79 (16.08-79.67)	2.04 (0.91-4.54)	2.04 (0.92-4.54)	2.13 (0.96-4.74)
	No	18.97 (18.45-19.50)	1 [Reference]	1 [Reference]	1 [Reference]
Levetiracetam in combination with any ASM	Yes	N/A[a]	N/A[a]	N/A[a]	N/A[a]
	No	18.97 (18.45-19.50)	1 [Reference]	1 [Reference]	1 [Reference]

© 2022 Vegrim HM et al. *JAMA Neurology*.

Hazard rates (HR) of cancer in offspring of women with prescription fills for antiseizure-medication (ASM) regardless of high dose folic acid prescriptions, stratified for maternal epilepsy and types and combinations of antiseizure medications.

Type of ASM	Filled prescription of ASM	Incidence rates per 100 000 person years	Crude HR, 95% CI	aHR1*, 95% CI	aHR2**, 95% CI
Lamotrigine monotherapy	Yes	29.19 (16.58-51.40)	1.44 (0.79-2.63)	1.43 (0.79-2.61)	1.47 (0.89-2.43)
	No	18.96 (18.44-19.50)	1 [Reference]	1 [Reference]	1 [Reference]
Lamotrigine in combination with any ASM	Yes	32.23 (20.03-51.84)	1.47 (0.89-2.42)	1.47 (0.89-2.42)	1.47 (0.89-2.42)
	No	18.95 (18.43-19.49)	1 [Reference]	1 [Reference]	1 [Reference]
Carbamazepine monotherapy	Yes	37.81 (16.99-84.17)	2.07 (0.93-4.64)	2.05 (0.92-4.59)	2.17 (0.97-4.86)
	No	18.97 (17.1-19.9)	1 [Reference]	1 [Reference]	1 [Reference]
Carbamazepine in combination with any ASM	Yes	30.04 (13.50-66.87)	1.69 (0.76-3.76)	1.67 (0.75-3.72)	1.77 (0.79-3.94)
	No	18.97 (18.45-19.51)	1 [Reference]	1 [Reference]	1 [Reference]
Other monotherapy[b]	Yes	N/A[a]	N/A[a]	N/A[a]	N/A[a]
	No	18.98 (18.46-19.52)	1 [Reference]	1 [Reference]	1 [Reference]

Abbreviations: aHR: adjusted hazard ratio; ASM: antiseizure medication; HR: hazard ratio
* aHR1: Adjustment for sex, maternal age and education
** aHR2: Including adjustment for maternal BMI, prior births with congenital anomalies, smoking during pregnancy, number of hospitalizations
Birth year, sex of the child and source country were applied as stratum for all models.
Analyses were performed without stratification on filled prescription for high dose folic acid to have sufficient number of exposed cancer cases to report HRs.

Superscript
[a] Not sufficient no. of exposed cancer cases to be reported.
[b] Other ASM: Topiramate, Clonazepam, Oxcarbazepine, Phenobarbital

© 2022 Vegrim HM et al. *JAMA Neurology*.

eTable 10. Effect of Maternal Epilepsy on Risk of Childhood Cancer

We stratified children born to women with and without epilepsy with no further stratification on whether they were exposed to high dose folic acid or anti-seizure medication, to examine any effect of maternal epilepsy. Three adjustment models were performed as described in the statistical analysis. We did not observe any association between maternal epilepsy and risk of childhood cancer in the offspring (aHR=1.0, 95% CI 0.7-1.4).

Association between maternal epilepsy and risk of childhood cancer in the offspring

Maternal epilepsy	Live births	Childhood cancer cases	Incidence rate per 100 000 person-years, 95% CI	Crude HR, 95% CI	aHR1, 95% CI[a]	aHR2, 95% CI[b]	aHR3, 95% CI[c]
Yes	27 784	48	23.5 (17.7-31.3)	1.2 (0.9-1.6)	1.2 (0.9-1.6)	1.0 (0.7-1.5)	1.0 (0.7-1.4)
No	3 351 387	4996	18.9 (18.4-19.5)	1 [Reference]	1 [Reference]	1 [Reference]	1 [Reference]

Abbreviations: aHR: adjusted hazard ratio; HR: hazard ratio
a) aHR1: Adjustment for maternal age and education
b) aHR2: Including adjustment for antiseizure medication exposure.
c) aHR3: Including adjustment for maternal BMI, prior births with congenital anomalies, smoking during pregnancy, number of hospitalizations.
Birth year, sex of the child and source country were applied as stratum for all models.

© 2022 Vegrim HM et al. *JAMA Neurology*.

eTable 11. Restrictions

For the main analysis in Table 2, showing risk of cancer in children born to mothers with epilepsy and with and without high dose folic acid exposure, we reran the analysis with separate restrictions on covariates that might have influenced the association. This included maternal cancer before pregnancy, maternal tuberous sclerosis, maternal diabetes mellitus one year before birth. Maternal fill for valproate or carbamazepine included both monotherapy and combination with other ASMs. We also included separate exclusions on children with major congenital anomaly and chromosomal abnormality.

Restriction by main analysis in Table 2: Association between maternal epilepsy, high dose folic acid and childhood cancer

**Restrictions**	**No. excluded from the analysis**	**Crude HR, 95% CI**	**aHR, 95% CI***
Maternal cancer before pregnancy	454	2.5 (1.3-4.6)	2.7 (1.2-6.3)
Maternal diagnosis of tuberous sclerosis	23	2.4 (1.3-4.5)	2.8 (1.2-6.3)
Maternal diabetes mellitus	1292	2.2 (1.2-4.1)	2.6 (1.1-5.9)
Maternal prescription fills for CBZ or VPA	3407	2.1 (0.9-4.5)	2.4 (0.9-6.5)
Major congenital anomaly in the child	1761	2.5 (1.3-5.0)	2.3 (1.0-5.7)
Chromosomal abnormalities in the child	68	2.6 (1.4-5.0)	2.7 (1.2-6.3)

Abbreviations: aHR: adjusted hazard ratio; ASM: antiseizure medication; CBZ: Carbamazepine; HR: hazard ratio; VPA: Valproate.
* aHR: Adjusting for maternal age- and education, maternal BMI, prior births with congenital anomalies, smoking during pregnancy, number of hospitalizations.

Birth year, sex of the child and source country were applied as stratum for all models.

© 2022 Vegrim HM et al. *JAMA Neurology*.

eTable 12. Definition of Included Covariates

Variable definitions

This table provide a complete overview with definitions on covariates and their sources that have been used in the paper "*High Dose Folic Acid during Pregnancy in Mothers with and without Epilepsy and Cancer Risk in their Children: A Scandinavian Register-based Cohort study*" by Vegrim et al.

The lay-out of the table is based on the S2 table from: "Relation of in-utero exposure to antiepileptic drugs to pregnancy duration and size at birth" by Margulis AV, Hernandez-Diaz H, McElrath T, Rothman KJ, Plana E, Almqvist C, D´Onofrio BM, Oberg AS.[1]

Contents

eTable 12 Definition of included covariates 14
- Maternal definitions 14
- Child definitions 16
- Definition of pregnancy complications 16
- Medical exposure definitions 17
- Maternal somatic comorbidities 19
- Childhood cancer definition according to the ICCC3 20

References 30

Abbreviations

ATC: Anatomical therapeutic chemical; CR: Cancer registers; BMI: Body mass index; ICCC3: International Classification of Childhood Cancer 3rd edition; ICD: International Classification of Diseases; ICD-O/3: International Classification of Diseases for Oncology, 3rd edition; LMP: Last menstrual period; MBR: Medical birth registers; NPR: National patient registers; NSA: National statistical agencies; N/A; Not applicable; PDR: Prescription registers.

© 2022 Vegrim HM et al. *JAMA Neurology*.

**eTable 12 Definition of included covariates**								
					**Codes**			
**Cohort characteristic**	**Time frame**	**Functional form of Variable and Comments**	**Register**	**Non-ICD or ATC-Coded Variables**	**ATC**	**ICD-10**	**ICD-O/3 Morphology**	**ICD-O/3 Topography**
**Maternal definitions**								
First day of last menstrual period (LMP).	N/A	Date of first day in last menstrual period. Estimated subtracting the date of birth from gestational age at birth.	MBR					
Maternal age.	At delivery.	Continuous.	MBR					
Maternal education.	Year of delivery.	Categorical:<br>0: Compulsory.<br>1: Pre-university.<br>2: College/University.<br>3: Post-graduate.<br>4: Missing information on education.	MBR					
Maternal country of birth.	N/A	Mother born in the country of delivery or not.<br>1) Source country.<br>2) All other countries.	NSA, MBR					
Calendar year of delivery.	Year of delivery	Continuous.	MBR					
Parity.	N/A	Number of previous deliveries: 0, 1 or ≥2.	MBR					
Child sex.	At delivery.	Categorical: Female or male.	MBR					

© 2022 Vegrim HM et al. *JAMA Neurology*.

**eTable 12 Definition of included covariates**								
					**Codes**			
**Cohort characteristic**	**Time frame**	**Functional form of Variable and Comments**	**Register**	**Non-ICD or ATC-Coded Variables**	**ATC**	**ICD-10**	**ICD-O/3 Morphology**	**ICD-O/3 Topography**
Child birthweight.	At delivery.	Continuous.	MBR					
Child gestational age.	At delivery	Continuous.	MBR					
BMI.	At start of pregnancy.	Categorical: As recorded in MBR or calculated from maternal weight and height at first antenatal visit (kg/m^2).<br>- Non-obese <35 kg/m^2.<br>- Obese ≥35 kg/m^2.<br>- Missing.	MBR					
Smoking.	Start of pregnancy.	Categorical: Y/N/Missing.	MBR	Checkbox MBR				
Previous congenital anomaly.	N/A	Categorial: Y/N. Computed as summarized previous births with congenital anomalies.	MBR					
Prior birth to child with major congenital anomaly.	Full lookback.	Categorical: Y/N.<br><br>Definition of major congenital malformation in accordance with EUROCAT definition[2].	NPR, MBR			Q00-Q99		

© 2022 Vegrim HM et al. *JAMA Neurology*.

**eTable 12 Definition of included covariates**								
					**Codes**			
**Cohort characteristic**	**Time frame**	**Functional form of Variable and Comments**	**Register**	**Non-ICD or ATC-Coded Variables**	**ATC**	**ICD-10**	**ICD-O/3 Morphology**	**ICD-O/3 Topography**
**Child definitions**								
Child sex.	At delivery.	Categorical: Female or male.	MBR					
Child birthweight.	At delivery.	Continuous.	MBR					
Child gestational age.	At delivery	Continuous.	MBR					
Chromosomal abnormality.	Any time.	Categorical: Y/N. Composite variable: Any of the codes for chromosomal abnormality.	NPR			Q90-Q99		
Congenital major anomaly	Any time.	Categorical: Y/N. Composite variable: Any of the codes for major congenital anomalies.<br><br>Definition of major congenital malformation in accordance with EUROCAT definition[2].	NPR			Q00-Q89		
**Definition of pregnancy complications**								
Gestational diabetes.	LMP-90 to delivery.		MBR, NPR	Checkbox MBR		O24.4 O24.9		

© 2022 Vegrim HM et al. *JAMA Neurology*.

eTable 12 Definition of included covariates								
					Codes			
**Cohort characteristic**	**Time frame**	**Functional form of Variable and Comments**	**Register**	**Non-ICD or ATC-Coded Variables**	**ATC**	**ICD-10**	**ICD-O/3 Morphology**	**ICD-O/3 Topography**
Gestational hypertension.	LMP-90 to delivery.	Any code for gestational hypertension, preeclampsia, HELLP and/or eclampsia.	MBR, NPR	Checkbox MBR		O11, O13-16		
**Medical exposure definitions**								
Any high dose folic acid.	LMP-90 to delivery.	Categorical: Y/N. Any filled prescriptions of either 1mg or 5mg.<br><br>Separate variables for filled prescriptions of 1mg or 5mg.	PDR		B03BB01			
Folic acid average dose.	LMP-90 to delivery.	Average daily dose of folic acid supplements calculated from the total amount of folic acid supplements dispensed, divided by the number within defined time frame.<br><br>Separate variables based on 1mg or 5mg only.	PDR		B03BB01<br>B03BB51			
Folic acid cumulative dose.	LMP-90 to delivery.	Cumulative folic acid dose based on total no. of pills dispensed during the defined time frame.<br><br>Separate variables based on 1mg or 5mg only.	PDR		B03BB01<br>B03BB51			
Any antiseizure medication.	LMP-90 to delivery.	Categorical: Y/N.<br><br>Any monotherapy or polytherapy	PDR		N03<br>N05BA09<br>S01EC01			

© 2022 Vegrim HM et al. *JAMA Neurology*.

**eTable 12 Definition of included covariates**								
					**Codes**			
**Cohort characteristic**	**Time frame**	**Functional form of Variable and Comments**	**Register**	**Non-ICD or ATC-Coded Variables**	**ATC**	**ICD-10**	**ICD-O/3 Morphology**	**ICD-O/3 Topography**
Valproate monotherapy.	LMP-90 to delivery.	Categorical: >1 prescriptions of valproate and no other type of ASM: Y/N.	PDR		N03AG01			
Lamotrigine monotherapy.	LMP-90 to delivery.	Categorical: >1 prescriptions of lamotrigine and no other type of ASM: Y/N.	PDR		N03AX09			
Levetiracetam monotherapy.	LMP-90 to delivery.	Categorical: >1 prescriptions of levetiracetam and no other type of ASM: Y/N.	PDR		N03AX14			
Carbamazepine monotherapy.	LMP-90 to delivery.	Categorical: >1 prescriptions of carbamazepine and no other type of ASM: Y/N.	PDR		N03AF01			
Topiramate monotherapy.	LMP-90 to delivery.	Categorical: >1 prescriptions: Y/N.	PDR		N03AX11			
Clonazepam monotherapy.	LMP-90 to delivery.	Categorical: >1 prescriptions: Y/N.	PDR		N03AE01			
Oxcarbazepine monotherapy.	LMP-90 to delivery.	Categorical: >1 prescriptions: Y/N.	PDR		N03AF02			
Phenobarbital monotherapy.	LMP-90 to delivery.	Categorical: >1 prescriptions: Y/N.	PDR		N03AB02			

© 2022 Vegrim HM et al. *JAMA Neurology*.

eTable 12 Definition of included covariates								
					Codes			
**Cohort characteristic**	**Time frame**	**Functional form of Variable and Comments**	**Register**	**Non-ICD or ATC-Coded Variables**	**ATC**	**ICD-10**	**ICD-O/3 Morphology**	**ICD-O/3 Topography**
Phenytoin monotherapy.	LMP-90 to delivery.	Categorical: ≥1 prescriptions: Y/N.	PDR		N03AB02			
**Maternal somatic comorbidities**								
Epilepsy.	Full lookback.	Categorical: Y/N	NPR, MBR, PDR	Check-box MBR		G40-41		
Diabetes mellitus.	LMP-365 to delivery.	Categorical: Y/N	NPR, MBR	Checkbox MBR		E10-14		
Tuberous sclerosis.	Full lookback.	Categorical: Y/N	NPR			Q85.1		
Number of hospitalizations.	LMP-365 to delivery.	Categorical: 0, 1, ≥2.	NPR					
Maternal cancer.	Any time until delivery.	Categorical: Y/N.	CR			C00-97	ICD-O/3 behavioral code of 3 except for CNS cancers.	Group based on ICD-10 C-chapters.

© 2022 Vegrim HM et al. *JAMA Neurology*.

eTable 12 Definition of included covariates								
					**Codes**			
**Cohort characteristic**	**Time frame**	**Functional form of Variable and Comments**	**Re gis ter**	**Non-ICD or ATC-Coded Variables**	**ATC**	**ICD-10**	**ICD-O/3 Morphology**	**ICD-O/3 Topography**
**Childhood cancer definition according to the ICCC3**								
I. Leukemias, myeloproliferative diseases and myelodysplastic diseases.								
Lymphoid leukemias.	Before age 20.	Categorical: Y/N. Composite variables: Any combination of specified ICD-O/3 morphology and topography codes.	CR				9820, 9823, 9826, 9827, 9831-9837, 9940, 9948	C000-C809
Acute myeloid leukemias.	Before age 20.	Categorical: Y/N. Composite variables: Any combination of specified ICD-O/3 morphology and topography codes.	CR				9840, 9861, 9866, 9867, 9870-9874, 9891, 9895-9897, 9910, 9920, 9931	C000-C809
I. Leukemias, myeloproliferative diseases and myelodyasplastic diseases: - *Chronic myeloproliferative diseases.*	Before age 20.	Categorical: Y/N. Composite variables: Any combination of specified ICD-O/3 morphology and topography codes.	CR				9863, 9875, 9876, 9950, 9960-9964	C000-C809
I. Leukemias, myeloproliferative diseases and myelodyasplastic diseases: - *Myelodysplastic syndrome and other myeloproliferative diseases.*	Before age 20.	Categorical: Y/N. Composite variables: Any combination of specified ICD-O/3 morphology and topography codes.	CR				9945, 9946, 9975, 9980, 9982-9987, 9989	C000-C809

© 2022 Vegrim HM et al. *JAMA Neurology*.

eTable 12 Definition of included covariates								
					Codes			
**Cohort characteristic**	**Time frame**	**Functional form of Variable and Comments**	**Re gis ter**	**Non-ICD or ATC-Coded Variables**	**ATC**	**ICD-10**	**ICD-O/3 Morphology**	**ICD-O/3 Topography**
I. Leukemias, myeloproliferative diseases and myelodyasplastic diseases:<br>- *Unspecified and other specified leukemias.*	Before age 20.	Categorical: Y/N. Composite variables: Any combination of specified ICD-O/3 morphology and topography codes.	CR				9800, 9801, 9805, 9860, 9930	C000-C809
II. Lymphomas and reticuloendothelial neoplasms.								
Hodgkin lymphomas.	Before age 20.	Categorical: Y/N. Composite variables: Any combination of specified ICD-O/3 morphology and topography codes.	CR				9650-9655, 9659, 9661-9665, 9667	C000-C809
Non-Hodgkin lymphomas (except Burkitt lymphoma).	Before age 20.	Categorical: Y/N. Composite variables: Any combination of specified ICD-O/3 morphology and topography codes.	CR				9591, 9670, 9671, 9673, 9675, 9678-9680, 9684, 9689-9691, 9695, 9698-9702, 9705, 9708, 9709, 9714, 9716-9719, 9727-9729, 9731-9734, 9760-9762, 9764-9769, 9970	C000-C809
Burkitt lymphoma**.**	Before age 20.	Categorical: Y/N. Composite variables: Any combination of specified ICD-O/3 morphology and topography codes.	CR				9687	C000-C809
Miscellaneous lymphoreticular neoplasms.	Before age 20.	Categorical: Y/N. Composite variables: Any combination of specified ICD-O/3 morphology and topography codes.	CR				9740-9742, 9750, 9754-9758	C000-C809

© 2022 Vegrim HM et al. *JAMA Neurology*.

eTable 12 Definition of included covariates								
					Codes			
**Cohort characteristic**	**Time frame**	**Functional form of Variable and Comments**	**Register**	**Non-ICD or ATC-Coded Variables**	**ATC**	**ICD-10**	**ICD-O/3 Morphology**	**ICD-O/3 Topography**
Unspecified lymphomas.	Before age 20.	Categorical: Y/N. Composite variables: Any combination of specified ICD-O/3 morphology and topography codes.	CR				9590, 9596	C000-C809
III. CNS and miscellaneous intracranial and intraspinal neoplasms.								
Ependymomas and choroid plexus tumor.	Before age 20.	Categorical: Y/N. Composite variables: Any combination of specified ICD-O/3 morphology and topography codes.	CR				9383, 9390-9394	C000-C809
Astrocytomas.	Before age 20.	Categorical: Y/N. Composite variables: Any combination of specified ICD-O/3 morphology and topography codes.	CR				9380<br><br>9384, 9400-9411, 9420, 9421-9424, 9440-9442	C723<br><br>C000-C809
Intracranial and intraspinal embryonal tumors.	Before age 20.	Categorical: Y/N. Composite variables: Any combination of specified ICD-O/3 morphology and topography codes.	CR				9470-9474, 9480, 9508<br><br>9501-9504	C000-C809<br><br>C700-C729
Other gliomas.	Before age 20.	Categorical: Y/N. Composite variables: Any combination of specified ICD-O/3 morphology and topography codes.	CR				9380<br><br>9381, 9382, 9430, 9444, 9450, 9451, 9460	C700-C722, C724-C729, C751, C753<br><br>C000-C809
Other specified intracranial and intraspinal neoplasms.	Before age 20.	Categorical: Y/N. Composite variables: Any combination of specified ICD-O/3 morphology and topography codes.	CR				8270-8281, 8300, 9350-9352, 9360-9362, 9412, 9413, 9492, 9493, 9505-9507, 9530-9539, 9582	C000-C809

© 2022 Vegrim HM et al. *JAMA Neurology*.

eTable 12 Definition of included covariates								
					Codes			
**Cohort characteristic**	**Time frame**	**Functional form of Variable and Comments**	**Re gis ter**	**Non-ICD or ATC-Coded Variables**	**ATC**	**ICD-10**	**ICD-O/3 Morphology**	**ICD-O/3 Topography**
Unspecified intracranial and intraspinal neoplasm.	Before age 20.	Categorical: Y/N. Composite variables: Any combination of specified ICD-O/3 morphology and topography codes.	CR				8000-8005	C700-C729, C751-C753
IV. Neuroblastoma and other peripheral nervous cell tumors.								
Neuroblastoma and ganglioneuroblastoma.	Before age 20.	Categorical: Y/N. Composite variables: Any combination of specified ICD-O/3 morphology and topography codes.	CR				9490, 9500	C000-C809
Other peripheral nervous cell tumors.	Before age 20.	Categorical: Y/N. Composite variables: Any combination of specified ICD-O/3 morphology and topography codes.	CR				8680-8683, 8690-8693, 8700, 9520-9523<br><br>9501-9504	C000-C809<br><br>C000-C699, C739-C768, C809
V. Retinoblastoma.								
Retinoblastoma.	Before age 20.	Categorical: Y/N. Composite variables: Any combination of specified ICD-O/3 morphology and topography codes.	CR				9510-9514	C000-C809
VI. Renal tumors.								

© 2022 Vegrim HM et al. *JAMA Neurology*.

eTable 12 Definition of included covariates								
					Codes			
**Cohort characteristic**	**Time frame**	**Functional form of Variable and Comments**	**Register**	**Non-ICD or ATC-Coded Variables**	**ATC**	**ICD-10**	**ICD-O/3 Morphology**	**ICD-O/3 Topography**
Nephroblastoma and other nonepithelial renal tumors.	Before age 20.	Categorical: Y/N. Composite variables: Any combination of specified ICD-O/3 morphology and topography codes.	CR				8959, 8960, 8964-8967<br><br>8963, 9364	C000-C809<br><br>C649
Renal carcinomas.	Before age 20.	Categorical: Y/N. Composite variables: Any combination of specified ICD-O/3 morphology and topography codes.	CR				8010-8041, 8050-8075, 8082, 8120-8122, 8130-8141, 8143, 8155, 8190-8201, 8210, 8211, 8221-8231, 8240, 8241, 8244-8246, 8260-8263, 8290, 8310, 8320, 8323, 8401, 8430, 8440, 8480-8490, 8504, 8510, 8550, 8560-8576<br><br>8311, 8312, 8316-8319, 8361	C649<br><br>C000-C809
Unspecified malignant renal tumors.	Before age 20.	Categorical: Y/N. Composite variables: Any combination of specified ICD-O/3 morphology and topography codes.	CR				C8000-8005	C649
VII. Hepatic tumors.								
Hepatoblastoma.	Before age 20.	Categorical: Y/N. Composite variables: Any combination of specified ICD-O/3 morphology and topography codes.	CR				8970	C000-C809

© 2022 Vegrim HM et al. *JAMA Neurology*.

eTable 12 Definition of included covariates								
					Codes			
**Cohort characteristic**	**Time frame**	**Functional form of Variable and Comments**	**Register**	**Non-ICD or ATC-Coded Variables**	**ATC**	**ICD-10**	**ICD-O/3 Morphology**	**ICD-O/3 Topography**
Hepatic carcinomas.	Before age 20.	Categorical: Y/N. Composite variables: Any combination of specified ICD-O/3 morphology and topography codes.	CR				8010-8041, 8050-8075, 8082, 8120-8122, 8140, 8141, 8143, 8155, 8190-8201, 8210, 8211, 8230, 8231, 8240, 8241, 8244-8246, 8260-8264, 8310, 8320, 8323, 8401, 8430, 8440, 8480-8490, 8504, 8510, 8550, 8560-8576<br><br>8160-8180	C220, C221<br><br>C000-C809
Unspecified malignant hepatic tumors.	Before age 20.	Categorical: Y/N. Composite variables: Any combination of specified ICD-O/3 morphology and topography codes.	CR				8000-8005	C220, C221
VIII. Malignant bone tumors.								
Osteosarcomas.	Before age 20.	Categorical: Y/N. Composite variables: Any combination of specified ICD-O/3 morphology and topography codes.	CR				9180-9187, 9191-9195, 9200	C400-C419, C760-C768, C809
Chondrosarcomas.	Before age 20.	Categorical: Y/N. Composite variables: Any combination of specified ICD-O/3 morphology and topography codes.	CR				9210, 9220, 9240<br><br>9221, 9230, 9241-9243	C400-C419, C760-C768, C809<br><br>C000-C809
Ewing tumor and related sarcomas of bone.	Before age 20.	Categorical: Y/N. Composite variables: Any combination of specified ICD-O/3 morphology and topography codes.	CR				9260<br><br>9363-9365	C400-C419, C760-C768, C809<br><br>C400-C419

© 2022 Vegrim HM et al. *JAMA Neurology*.

**eTable 12 Definition of included covariates**								
					**Codes**			
**Cohort characteristic**	**Time frame**	**Functional form of Variable and Comments**	**Register**	**Non-ICD or ATC-Coded Variables**	**ATC**	**ICD-10**	**ICD-O/3 Morphology**	**ICD-O/3 Topography**
Other specified malignant bone tumors.	Before age 20.	Categorical: Y/N. Composite variables: Any combination of specified ICD-O/3 morphology and topography codes.	CR				8810, 8811, 8823, 8830<br><br>8812, 9250, 9261, 9262, 9270-9275, 9280-9282, 9290, 9300-9302, 9310-9312, 9320-9322, 9330, 9340-9342, 9370-9372	C400-C419<br><br>C000-C809
Unspecified malignant bone tumors.	Before age 20.	Categorical: Y/N. Composite variables: Any combination of specified ICD-O/3 morphology and topography codes.	CR				8000-8005, 8800, 8801, 8803-8805	C400-C419
IX. Soft tissue and other extraosseous sarcomas.								
Rhabdomyosarcomas.	Before age 20.	Categorical: Y/N. Composite variables: Any combination of specified ICD-O/3 morphology and topography codes.	CR				8900-8905, 8910, 8912, 8920, 8991	C000-C809
Fibrosarcomas, peripheral nerve sheath tumors and other fibrous neoplasms.	Before age 20.	Categorical: Y/N. Composite variables: Any combination of specified ICD-O/3 morphology and topography codes.	CR				8810, 8811, 8813-8815, 8821, 8823, 8834-8835<br><br>8820, 8822, 8824-8827, 9150, 9160, 9491, 9540-9571, 9580	C000-C399, C440-C768, C809<br><br>C000-C809
Kaposi sarcoma.	Before age 20.	Categorical: Y/N. Composite variables: Any combination of specified ICD-O/3 morphology and topography codes.	CR				9140	C000-C809

© 2022 Vegrim HM et al. *JAMA Neurology*.

eTable 12 Definition of included covariates								
					Codes			
Cohort characteristic	Time frame	Functional form of Variable and Comments	Re gis ter	Non-ICD or ATC-Coded Variables	ATC	ICD-10	ICD-O/3 Morphology	ICD-O/3 Topography
Other specified soft tissue sarcomas.	Before age 20.	Categorical: Y/N. Composite variables: Any combination of specified ICD-O/3 morphology and topography codes.	CR				8587, 8710-8713, 8806, 8831-8833, 8836, 8840-8842, 8850-8858, 8860-8862, 8870, 8880, 8881, 8890-8898, 8921, 8982, 8990, 9040-9044, 9120-9125, 9130-9133, 9135, 9136, 9141, 9142, 9161, 9170-9175, 9231, 9251, 9252, 9373, 9581<br>8830<br>8963<br>9180, 9210, 9220, 9240<br>9260<br>9364<br>9365	C000-C809<br>C000-C399, C440-C768, C809<br>C000-C639, C659-C699, C739-C768, C809<br>C490-C499<br>C000-C399, C470-C759<br>C000-C399, C470-C639, C659-C699, C739-C768, C809<br>C000-C399, C470-C639, C659-C768, C809
Unspecified soft tissue sarcomas.	Before age 20.	Categorical: Y/N. Composite variables: Any combination of specified ICD-O/3 morphology and topography codes.	CR				8800-8805	C000-C399, C440-C768, C809
X. Germ cell tumors, trophoblastic tumors and neoplasms of gonads.								

© 2022 Vegrim HM et al. *JAMA Neurology*.

**eTable 12 Definition of included covariates**								
					**Codes**			
**Cohort characteristic**	**Time frame**	**Functional form of Variable and Comments**	**Register**	**Non-ICD or ATC-Coded Variables**	**ATC**	**ICD-10**	**ICD-O/3 Morphology**	**ICD-O/3 Topography**
Intracranial and intraspinal germ cell tumors.	Before age 20.	Categorical: Y/N. Composite variables: Any combination of specified ICD-O/3 morphology and topography codes.	CR				9060-9065, 9070-9072, 9080-9085, 9100, 9101	C700-C729, C751-C753
Malignant extracranial and extragonadal germ cell tumors.	Before age 20.	Categorical: Y/N. Composite variables: Any combination of specified ICD-O/3 morphology and topography codes.	CR				9060-9065, 9070-9072, 9080-9085, 9100-9105	C000-C559, C570-C619, C630-C699, C739-C750, C754-C768, C809
Malignant gonadal germ cell tumors.	Before age 20.	Categorical: Y/N. Composite variables: Any combination of specified ICD-O/3 morphology and topography codes.	CR				9060-9065, 9070-9073, 9080-9085, 9090, 9091, 9100, 9101	C569, C620-C629
Gonadal carcinomas.	Before age 20.	Categorical: Y/N. Composite variables: Any combination of specified ICD-O/3 morphology and topography codes.	CR				8010-8041, 8050-8075, 8082, 8120-8122, 8130-8141, 8143, 8190-8201, 8210, 8211, 8221-8241, 8244-8246, 8260-8263, 8290, 8310, 8313, 8320, 8323, 8380-8384, 8430, 8440, 8480-8490, 8504, 8510, 8550, 8560-8573, 9000, 9014, 9015<br><br>8441-8444, 8450, 8451, 8460-8473	C569, C620-C629<br><br>C000-C809
Other and unspecified malignant gonadal tumors.	Before age 20.	Categorical: Y/N. Composite variables: Any combination of specified ICD-O/3 morphology and topography codes.	CR				8590-8671<br><br>8000-8005	C000-C809<br><br>C569, C620-C629
XI. Other malignant epithelial neoplasms and malignant melanomas.								

© 2022 Vegrim HM et al. *JAMA Neurology*.

eTable 12 Definition of included covariates								
					Codes			
**Cohort characteristic**	**Time frame**	**Functional form of Variable and Comments**	**Register**	**Non-ICD or ATC-Coded Variables**	**ATC**	**ICD-10**	**ICD-O/3 Morphology**	**ICD-O/3 Topography**
Adrenocortical carcinomas.	Before age 20.	Categorical: Y/N. Composite variables: Any combination of specified ICD-O/3 morphology and topography codes.	CR				8370-8375	C000-C809
Thyroid carcinomas.	Before age 20.	Categorical: Y/N. Composite variables: Any combination of specified ICD-O/3 morphology and topography codes.	CR				8010-8041, 8050-8075, 8082, 8120-8122, 8130-8141, 8190, 8200, 8201, 8211, 8230, 8231, 8244-8246, 8260-8263, 8290, 8310, 8320, 8323, 8430, 8440, 8480, 8481, 8510, 8560-8573<br><br>8330-8337, 8340-8347, 8350	C739<br><br>C000-C809
Nasopharyngeal carcinomas.	Before age 20.	Categorical: Y/N. Composite variables: Any combination of specified ICD-O/3 morphology and topography codes.	CR				8010-8041, 8050-8075, 8082, 8083, 8120-8122, 8130-8141, 8190, 8200, 8201, 8211, 8230, 8231, 8244-8246, 8260-8263, 8290, 8310, 8320, 8323, 8430, 8440, 8480, 8481, 8500-8576	C110-C119
Malignant melanomas.	Before age 20.	Categorical: Y/N. Composite variables: Any combination of specified ICD-O/3 morphology and topography codes.	CR				8720-8780, 8790	C000-C809
Skin carcinomas.	Before age 20.	Categorical: Y/N. Composite variables: Any combination of specified ICD-O/3 morphology and topography codes.	CR				8010-8041, 8050-8075, 8078, 8082, 8090-8110, 8140, 8143, 8147, 8190, 8200, 8240, 8246, 8247, 8260, 8310, 8320, 8323, 8390-8420, 8430, 8480, 8542, 8560, 8570-8573, 8940, 8941	C440-C449

© 2022 Vegrim HM et al. *JAMA Neurology*.

**eTable 12 Definition of included covariates**								
					**Codes**			
**Cohort characteristic**	**Time frame**	**Functional form of Variable and Comments**	**Register**	**Non-ICD or ATC-Coded Variables**	**ATC**	**ICD-10**	**ICD-O/3 Morphology**	**ICD-O/3 Topography**
Other and unspecified carcinomas.	Before age 20.	Categorical: Y/N. Composite variables: Any combination of specified ICD-O/3 morphology and topography codes.	CR				8010-8084, 8120-8157, 8190-8264, 8290, 8310, 8313-8315, 8320-8325, 8360, 8380-8384, 8430-8440, 8452-8454, 8480-8586, 8588-8589, 8940, 8941, 8983, 9000, 9010-9016, 9020, 9030	C000-C109, C129-C218, C239-C399, C480-C488, C500-C559, C570-C619, C630-C639, C659-C729, C750-C768, C809
XII. Other and unspecified malignant neoplasms.								
Other specified malignant tumors.	Before age 20.	Categorical: Y/N. Composite variables: Any combination of specified ICD-O/3 morphology and topography codes.	CR				8930-8936, 8950, 8951, 8971-8981, 9050-9055, 9110<br><br>9363	C000-C809<br><br>C000-C399, C470-C759
Other unspecified malignant tumors.	Before age 20.	Categorical: Y/N. Composite variables: Any combination of specified ICD-O/3 morphology and topography codes.	CR				8000-8005	C000-C218, C239-C399, C420-C559, C570-C619, C630-C639, C659-C699, C739-C750, C754-C809

eReferences.

1. Margulis AV, Hernandez-Diaz S, McElrath T, et al. Relation of in-utero exposure to antiepileptic drugs to pregnancy duration and size at birth. *PLoS One.* 2019;14(8):e0214180.
2. EUROCAT. EUROCAT Guide 1.4 and Reference Documents 2018.

© 2022 Vegrim HM et al. *JAMA Neurology*.
